# Regulation of Selective B Cell Autophagy by the Pro-oxidant Adaptor p66SHC

**DOI:** 10.3389/fcell.2020.00193

**Published:** 2020-03-26

**Authors:** Anna Onnis, Chiara Cassioli, Francesca Finetti, Cosima T. Baldari

**Affiliations:** Department of Life Sciences, University of Siena, Siena, Italy

**Keywords:** p66SHC, autophagy, mitophagy, B lymphocytes, ROS

## Abstract

p66SHC is a pro-oxidant member of the SHC family of protein adaptors that acts as a negative regulator of cell survival. In lymphocytes p66SHC exploits both its adaptor and its reactive oxygen species (ROS)-elevating function to antagonize mitogenic and survival signaling and promote apoptosis. As a result, p66SHC deficiency leads to the abnormal expansion of peripheral T and B cells and lupus-like autoimmunity. Additionally, a defect in p66SHC expression is a hallmark of B cell chronic lymphocytic leukemia, where it contributes to the accumulation of long-lived neoplastic cells. We have recently provided evidence that p66SHC exerts a further layer of control on B cell homeostasis by acting as a new mitochondrial LC3-II receptor to promote the autophagic demise of dysfunctional mitochondria. Here we discuss this finding in the context of the autophagic control of B cell homeostasis, development, and differentiation in health and disease.

## Introduction

Initially reported as a longevity-related protein, the p66 kDa isoform of SHC1, a member of the SHC protein family, is a ubiquitously expressed protein that participates in a plethora of pathways through its ability to act both as an adapter that antagonizes tyrosine kinase-dependent mitogenic signaling and as a pro-oxidant molecule molecule that promotes stress-dependent apoptosis. The reactive oxygen species (ROS)-elevating activity of p66SHC has been recently linked to other cellular functions, including inhibition of anabolic metabolism and expression of ROS-sensitive genes (Nemoto and Finkel, 2002; Nemoto et al., 2006; Soliman et al., 2014). Similar to other cell types, p66SHC attenuates mitogenic and survival signaling triggered by the antigen receptors in T and B lymphocytes and promotes their apoptotic demise (Finetti et al., 2009). p66SHC also inhibits chemokine receptor signaling in B cells at multiple levels, thereby impinging on their traffic to the pro-survival lymphoid tissues. Not surprisingly, p66SHC deficiency is associated with pathogenic outcomes, including autoimmunity and leukemia (Finetti et al., 2008; Capitani et al., 2010; Patrussi et al., 2019). We have recently reported a new role for p66SHC as regulator of B-cell autophagy/mitophagy and differentiation (Onnis et al., 2018). Here we will summarize our current understanding of the multifaceted role of p66SHC in lymphocyte survival and discuss the pro-autophagic/mitophagic function of p66SHC in the context of the autophagic control of B-cell development and differentiation.

## p66Shc: a Master Regulator of Cell Survival

**FIGURE 1:**
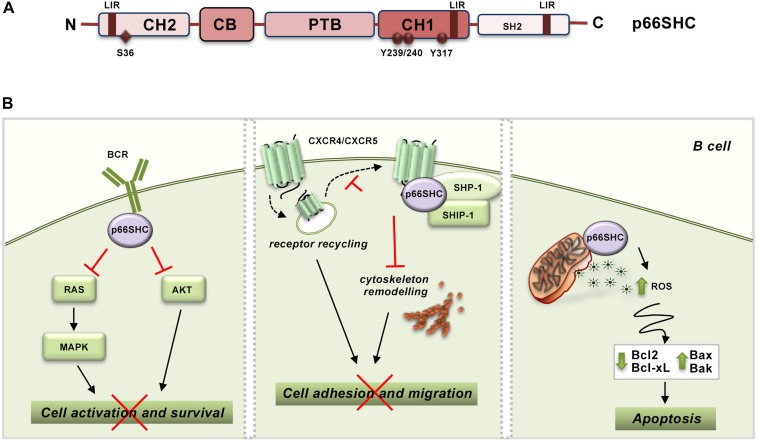
p66SHC structure and functions in B lymphocytes. **(A)** Schematic representation of the modular organization of p66SHC. The scheme shows the N-terminal collagen-homology domain (CH2), the CYCS-binding domain (CB), the phosphotyrosine-binding domain (PTB), the collagen homology domain (CH1), and the C-terminal SRC-homology domain 2 (SH2) together with the phosphorylatable tyrosine and serine residues, and the LIR motifs involved in the interaction with LC3B. **(B)** Role of p66SHC in BCR and chemokine signaling and apoptosis in B lymphocytes. *Left panel*, After BCR engagement p66SHC inhibits cell activation and survival by impairing Ras/MAPK and Akt activation. *Middle panel*, p66SHC promotes the assembly of a complex with the phosphatases SHP-1 and SHIP-1 on CXCR4 or CXCR5 which inhibits cytoskeleton remodeling and hence B cell adhesion and migration. In addition, p66SHC decreases the surface levels of these chemokine receptors slowing down their recycling to the plasma membrane (dotted lines), and, as a result, negatively regulates their ability to signal in B lymphocytes. *Right panel*, p66SHC increases B cell susceptibility to apoptosis by inducing production of ROS and modulating the expression of several members of the BCL-2 family of apoptosis-regulating proteins. The wide-ranging functions of p66Shc depicted in this scheme, which are also present in T cells, are associated to pathogenic outcomes. Indeed, p66Shc deficiency is a causal factor for the development of lupus-like autoimmunity in mice and for the onset and severity of human CLL.

The function of SHC proteins as molecular adapters relies on their modular structure, consisting of a N-terminal phosphotyrosine binding (PTB) domain, a proline- and glycine-rich collagen homology (CH1) domain that includes three phoshorylatable tyrosine residues, and a C-terminal SRC-homology (SH2) domain. p66SHC is the largest of the three isoforms encoded by SHC1. While sharing the PTB-CH1-SH2 modular structure with the other isoforms, p52SHC and p46SHC, p66SHC is characterized by an additional N-terminal collagen–homology domain (CH2) containing a phosphorylatable serine residue at position 36 (S36) and a cytochrome c, somatic (CYCS) binding domain mapping to the N-terminal end of the PTB domain. While the latter is shared with p52SHC, the CYCS binding ability is very low in this isoform (approximately 10% compared to p66SHC) (Giorgio et al., 2005; Figure 1A). These molecular determinants confer p66SHC unique properties compared to p52SHC, the prototype SHC adapter initially implicated in coupling the EGF receptor to RAS activation through its ability to locally recruit the adapter GRB2 and the RAS guanine nucleotide exchanger, SOS (Pelicci et al., 1992; Pronk et al., 1994; Sasaoka et al., 1994). Indeed, when phosphorylated on S36 p66SHC acts a competitive inhibitor of p52SHC, preventing its recruitment to activated receptors and, as a result, inhibiting mitogenic as well as survival signaling (Migliaccio et al., 1997; Okada et al., 1997). Additionally, p66SHC promotes apoptosis independently of its adapter function. Under conditions of cellular stress, which induce S36 phosphorylation, p66SHC translocates to the mitochondrial intermembrane space (IMS) with the assistance of the prolyl *cis*-trans isomerase PIN1 (Pinton et al., 2007). There it acts as a redox enzyme, inducing mitochondrial H_2_O_2_ production by transferring electrons from reduced CYCS to molecular oxygen (Giorgio et al., 2005). p66SHC enhances moreover the accumulation of intracellular ROS indirectly through its ability to inhibit FOXO transcription factors, which control the expression of enzymes involved in oxidant scavenging (Nemoto and Finkel, 2002).

### p66SHC Regulates Survival Signaling and Apoptosis in Lymphocytes

At variance with p52SHC and p46SHC that are constitutively and ubiquitously expressed, the expression of p66SHC is regulated by an alternative promoter in *ShcA/Shc1*. p66SHC expression is controlled epigenetically by methylation of a CpG island within the gene promoter, transcriptionally by STAT4 activity and post-translationally by p53-dependent protein stabilization (Trinei et al., 2002; Ventura et al., 2002; Cattaneo et al., 2016). Hence p66SHC is expressed at different levels in different tissues, with obvious implications to its ability to competitively inhibit p52SHC-mediated signaling. In lymphocytes, p66SHC expression is relatively low compared to p52SHC, although it is enhanced in response to cellular stress (Pelicci et al., 1992; Pezzicoli et al., 2006). In these cells, p66SHC exerts wide-ranging effects. p66SHC effectively inhibits T cell (TCR) and B cell (BCR) antigen receptor signaling both *in vitro* and *in vivo* (Pacini et al., 2004; Capitani et al., 2010). Both early and late signals triggered by the TCR and BCR are tuned down by p66SHC, indicating that it participates at the earliest steps in the respective signaling cascades (Pacini et al., 2004; Capitani et al., 2010). By acting as an early negative regulator of antigen receptor signaling, p66SHC impairs not only RAS/MAPK-dependent mitogenic signaling, but also survival signaling mediated by the phosphatidylinositol-3 kinase effector AKT (Capitani et al., 2010; Figure 1B). Consistent with this function, T and B cells from *p66shc^–/–^* mice show increased spontaneous and antigen-induced activation, proliferation and survival (Finetti et al., 2008).

Interestingly, p66SHC is also implicated as a negative regulator in both chemotactic and survival signaling by the chemokine receptor CXCR4 (Patrussi et al., 2014), which can be accounted for, at least in part, by the fact that CXCR4 is able to transactivate the TCR (Kumar et al., 2006; Patrussi et al., 2007). In B cells p66SHC exploits the phosphorylatable tyrosine residues in the CH1 domain not only to competitively inhibit p52SHC but also to promote the assembly of an inhibitory complex on CXCR4 and the related homing receptor CXCR5. This complex, which includes the phosphatases SHP-1 (Src homology phosphatase-1) and SHIP-1 (SH2 domain-containing inositol 5′-phosphatase-1), impairs actin cytoskeleton reorganization in response to CXCR4 or CXCR5 engagement, which limits B cell adhesion to integrin ligands and migration toward the respective chemokines (Patrussi et al., 2014). Additionally, in B cells p66SHC slows down recycling to the plasma membrane of the chemokine receptors CXCR4 and CCR7, which results in a decrease in their surface levels, by preventing the Ca^2+^-dependent transit of internalized receptors from early to recycling endosomes (Patrussi et al., 2018; Figure 1B). Since lymphocytes acquire survival signals during their cyclic traffic through secondary lymphoid organs, the modulation of chemokine receptor signaling by p66SHC at multiple steps contributes to its ability to negatively regulate lymphocyte survival.

In addition to its ability to inhibit survival signaling at multiple levels, p66SHC increases the susceptibility of lymphocytes to cellular stress, promoting apoptosis (Pellegrini et al., 2007; Capitani et al., 2010). Pharmacological or physiological apoptotic stimuli induce p66SHC phosphorylation on S36 through a mechanism requiring Ca^2+^ calmodulin-dependent kinase and the tyrosine kinase LCK (Pacini et al., 2004; Patrussi et al., 2012). S36-phosphorylated p66SHC promotes apoptosis by impairing both mitochondrial function and Ca^2+^ homeostasis (Pellegrini et al., 2007). The mechanisms underlying these activities have been in part elucidated. p66SHC has been shown to facilitate the dissipation of the mitochondrial transmembrane potential through its ROS-elevating activity, which results in a decrease in ATP production and eventually CYCS release (Trinei et al., 2002; Giorgio et al., 2005). We have additionally causally associated the disrupting effects of p66SHC on mitochondrial function to its ability to modulate the expression of several members of the BCL-2 family of apoptosis-regulating proteins (Pacini et al., 2004; Capitani et al., 2010; Figure 1B). This property can also account for the Ca^2+^-elevating activity of p66SHC, which we found associated with a decrease in the levels of the plasma membrane Ca^2+^ ATPase 4. This defect results in the inability of cells to extrude Ca^2+^ ions, leading to Ca^2+^ overload and apoptosis (Pellegrini et al., 2007).

### Pathogenic Outcomes of p66SHC Deficiency in Lymphocytes

Consistent with the central role played by p66Shc in the regulation of lymphocyte activation, survival and apoptosis, p66SHC deficiency is associated to the breaking of immunologic tolerance. Indeed, *p66Shc*^–/^*^–^* mice show increased spontaneous lymphocyte activation and proliferation, production of anti-dsDNA autoantibodies, and deposition of immune complexes in kidney and skin. This leads to the age-related development of lupus-like autoimmunity characterized by glomerulonephritis and alopecia (Finetti et al., 2008).

p66SHC deficiency is also as a causal factor in the development and severity of B cell chronic lymphocytic leukemia (B-CLL) (Capitani et al., 2010; Patrussi et al., 2019). B-CLL is the most common B cell neoplasm in the Western world, characterized by the accumulation of long-lived leukemic B cells in blood, bone marrow and secondary lymphoid organs (Hallek et al., 2018). CLL B cells have a defect in p66SHC expression, the severity of which correlates with unfavorable prognosis, that prevents their apoptosis and increases their chemioresistance (Capitani et al., 2010). This feature results from the ability of p66SHC to modulate the expression of BCL-2 family members through its ROS-elevating activity, such that its deficiency leads to an imbalance in the BCL-2 family toward survival. Additionally, p66SHC deficiency prolongs leukemic cell survival through its ability to control B cell homing to secondary lymphoid organs by limiting surface expression and signaling by homing receptors (see section “p66SHC Regulates Survival Signaling and Apoptosis in Lymphocytes”). The pro-oxidant activity of p66SHC is also implicated in this function as it is able to modulate in opposite directions the expression of the homing receptor CCR7 and of the sphingosine-1 phosphate receptor S1PR1, which drives B cell exit from lymphoid tissues. Excessive expression and signaling by homing receptors, together with defective expression of S1PR1, favor the residency of the p66SHC-deficient leukemic cells in the pro-survival and chemioprotective lymphoid niche (Capitani et al., 2010; Patrussi et al., 2018, 2019).

As we will discuss in the following sections, we have identified a new survival-related function of p66Shc as regulator of B lymphocyte autophagy (Onnis et al., 2018), a process that regulates B cell fate, survival and differentiation, highlighting p66SHC as a pleiotropic master regulator of lymphocyte survival.

## The Multifaceted Role of Autophagy in B Cells

Autophagy is a highly conserved catabolic process that allows for recycling of cytoplasmic proteins and organelles. Macroautophagy, microautophagy and chaperone-mediated autophagy (CMA) are the three main autophagic pathways described in mammals (Parzych and Klionsky, 2014). They differ in regulation, type of cargo and mechanism by which autophagic substrates are targeted to the lysosome for degradation. During micro- and macro-autophagy cytosolic components undergo in-bulk sequestration. In the former this occurs through invaginations of the lysosomal membrane, while the latter depends on the formation of new, double membrane vesicles, known as autophagosomes, that eventually fuse with the lysosome allowing for degradation of their contents. Conversely, soluble cytosolic proteins carrying the KFERQ-motif are typical substrates of CMA and their transfer into the lysosomal lumen requires the cooperation of a cytosolic chaperone with a receptor localized at the lysosomal membrane. Although the cell coordinates the different pathways in order to fulfill cellular demands under various conditions, the execution of each single pathway is under the control of specific regulators, which have been identified in autophagy-related (ATG) proteins for macroautophagy, or signaling events that results in changes of the lysosomal membrane composition required for CMA. Molecular details of microautophagy in mammals remain largely unknown, because homologs of the yeast genes have not been identified as yet.

**FIGURE 2:**
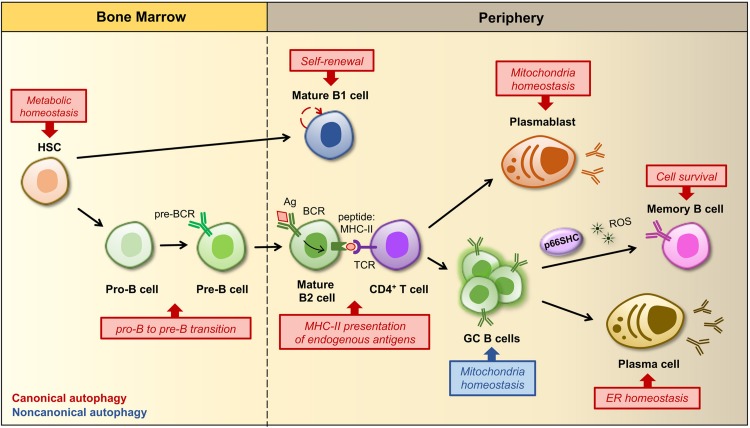
Regulation of B cell development, activation and differentiation by autophagy. B cells develop and differentiate into an array of peripheral B cell subsets through a stepwise process starting from a hematopoietic stem cell (HSC). The figure shows a simplified outline of B cell lineage differentiation with a particular focus on the stages in which autophagy and mitochondria have been implicated in the regulation of B cell survival, fate and/or functions. In the bone marrow autophagy maintains the metabolism of HSCs by preventing the accumulation of mitochondrial ROS, which regulate their self-renewal and differentiation (Ho et al., 2017). The HSC gives rise to progenitors of two distinct populations of B lymphocytes, called B1 and B2 cells. B1 B cell generation is restricted to fetal and early neonatal life and thereafter a population of B1 B cells is preserved in the periphery by self-renewal. The ability of B1 B cells to self-renew is dependent on autophagy and its role in the regulation of cellular metabolism as well as lipid and mitochondrial homeostasis. Conversely, the production of B2 B cells is constant throughout life, thus autophagy is dispensable for their survival and self-renewal, but required at the pro-B to pre-B transition, when immunoglobulin gene rearrangements lead to the expression of a pre-B cell receptor (BCR) on the cell surface. Within secondary lymphoid tissues (i.e., lymph nodes and spleen) activation of a mature B cell and its subsequent differentiation into an antibody-secreting plasma cell or a memory B cell requires the specific recognition of a foreign antigen (Ag) by the BCR and critical signals derived from an antigen-specific CD4^+^ T cell. After Ag-BCR binding, the Ag undergoes receptor-dependent internalization and delivered to the endosomal compartment, where it is degraded into peptides, then associated to MHC-II molecules and transferred back to the surface, where it is presented to a matched helper T cell. Autophagy participates in antigen presentation by favoring the degradation and modification of foreign antigens. Some activated B cells develop into short-lived plasmablasts that rely on autophagy for pruning mitochondrial content, while others proliferate within a germinal center (GC), where an increased non-canonical autophagy has been implicated in the control of mitochondrial mass and ROS production. Since ROS act as signaling molecules for B cell fate, autophagy and related changes in mitochondrial content and ROS levels are crucial for driving GC cell differentiation. At later stages autophagy limits endoplasmic reticulum (ER) stress caused by the robust antibody secretory activity of plasma cells, while it favors the maintenance of immunological memory against viral infections by protecting memory B cells from oxidative stress and lipid peroxidation toxicity. In this scenario, p66SHC participates in the regulation of B cell fate by promoting the differentiation of activated B cells to isotype-switched memory cells through its mitochondria-depolarizing and ROS-generating activity.

Macroautophagy (hereafter autophagy) is the most extensively studied form of autophagy and probably the one that contributes, to a major extent, to the lysosome-dependent degradation of autophagic cargo inside the cell. Therefore, it is not surprising that, similar to other cell types, lymphocytes exploit autophagy to preserve cellular homeostasis (McLeod et al., 2012). In addition to an essential quality control function, autophagic degradation of macromolecules provides energy and building blocks for synthesis of new molecules accounting, at least in part, for the metabolic plasticity of lymphocytes and hence their capacity of adapting to multiple environmental conditions over their lifetime. Focusing on B lymphocytes, autophagy has now emerged as crucial for the maintenance of certain B cell populations (i.e., memory B cells and plasma cells), while its role in the development and survival of naïve B cells remains largely to be elucidated. Moreover, at precise steps of the life of a B cell autophagy prevents endoplasmic reticulum (ER) stress and also participates in antigen presentation by B cells, a key event that allows a B cell to receive help from a T cell to eventually differentiate into a specialized effector cell (Arbogast and Gros, 2018; Sandoval et al., 2018). Autophagy also contributes to B cell homeostasis by preserving, among other things, a pool of healthy mitochondria and thus limiting ROS production (Figure 2). Although mitochondria can be enclosed into the autophagosome in a non-selective manner by autophagy, damaged or unwanted mitochondria are selectively removed by mitophagy, in which the core autophagic machinery is co-opted for mitochondria clearance (Ashrafi and Schwarz, 2013). Within the general framework of the autophagic process, cargo specificity is achieved with the assistance of LC3-interacting proteins that directly bridge the autophagosome membrane to dysfunctional mitochondria, thus promoting the execution of mitophagy.

### Autophagy in B Cell Homeostasis, Development and Differentiation

Current evidence points to autophagy as a master regulator of specific steps in the process that, starting from B cell development in the bone marrow, leads to the generation of humoral immunity and immunological memory. Autophagy has been implicated in the survival of developing B cells at the transition from the pro-B to the pre-B stage (Miller et al., 2008). During this step recombination at the immunoglobulin heavy chain locus occurs, allowing for the expression of the pre-BCR on which progression through the subsequent developmental program crucially depends (Melchers, 2015). As such autophagy plays an essential role in the generation of mature B cells, even though it cannot be excluded that defects in B cell development might reflect an earlier function of autophagy in protecting hematopoietic stem cells (HSC) from metabolic stress (Mortensen et al., 2011; Warr et al., 2013).

The role of autophagy in naïve B cell activation is as yet debated. Chen et al. (2014) have reported that autophagy is dispensable for the generation of a primary antiviral immune response. However, more recently Martinez-Martin and collaborators demonstrated that autophagy is active at the highest rate in germinal center (GC) B cells, suggesting that the autophagic activity may have an impact on certain activated stages during B cell responses (Martinez-Martin et al., 2017). Moreover, they observed that in both GC B cells and stimulated naïve B cells LC3-II accumulation occurs through a non-canonical pathway and is further enhanced in the absence of WD repeat domain, phosphoinositide-interacting protein 2 (WIPI2). Interestingly, B cells genetically depleted of WIPI2 not only showed increased non-canonical autophagy, but were also characterized by alterations in mitochondrial mass, mitochondrial membrane potential and ROS production. Additionally, during B cell activation the mitochondrial status and ROS function as signals that regulate B cell fate by favoring either class-switch recombination (CSR) or plasma cell differentiation (PCD) (Jang et al., 2015). Therefore, the impact of a temporal switch from canonical to non-canonical autophagy goes beyond a simple metabolic change, extending to mitochondrial homeostasis and B cell differentiation. Autophagy was also found to promote B1 cell survival and to support their ability to self-renew (Miller et al., 2008). In this particular case, the autophagic process contributes to maintain an active metabolic state required for self-renewal, which is characterized by high rates of lipid storage, glycolysis and mitochondrial oxidative phosphorylation (Clarke et al., 2018), as witnessed by a selective loss of B1 cells in the periphery when autophagy genes are deleted (Miller et al., 2008).

At variance with naïve B cells, the role of autophagy at late stages of B cell differentiation is well established (Conway et al., 2013). Memory B cells display high levels of basal autophagy and an enhanced expression of genes that encode components of the autophagic machinery. *In vivo* studies aimed at assessing the immune response in mice with ATG7-depleted B cells immunized against influenza virus and the challenged with the virus showed that only secondary responses were compromised in the absence of autophagy (Chen et al., 2014). This suggests that autophagy is likely to be required not so much for the generation as for the maintenance of memory B cells that become rapidly activated and ensure a robust protection during secondary immune responses. An explanation for the critical role of autophagy in the maintenance of immunological memory against influenza virus infection is that long-lived B cells have an increased probability to accumulate dysfunctional organelles which would lead to their apoptotic demise, such that autophagy would protect them from oxidative stress and lipid peroxidation toxicity (Chen et al., 2014). Besides memory B cells, plasma cells also rely on autophagy. During their differentiation, plasma cells undergo a substantial expansion of the ER network, required for sustained antibody synthesis. Therefore, they are continuously exposed to high levels of ER stress and exploit a selective form of autophagy, known as ER-phagy, to trim the ER and limit their secretory activity in favor of cell survival (Pengo et al., 2013). In addition to ER homeostasis, autophagy also contributes to plasmablast survival after lipopolysaccharide stimulation by limiting potential damages derived from an increased number of dysfunctional mitochondria (Arnold et al., 2016).

### Autophagy Is Implicated in Antigen Presentation by B Cells

Despite their primary role in humoral immunity, B cells function as antigen-presenting cells (APCs) in T cell-dependent responses in order to prime helper T cells, which in turn provide instructive signals for their terminal differentiation into memory or plasma cells secreting high affinity antibodies. Generally, antigenic peptides derived from extracellular proteins are loaded onto major histocompatibility class II (MHC-II) molecules for presentation. However, B cells can also efficiently process endogenous antigens that are presented to CD4^+^ T cells in association with MHC-II molecules. Multiple pathways, including CMA and autophagy, have been implicated in the delivery of cytosolic and nuclear antigens to the lysosome for their eventual presentation on MHC-II molecules. Starting from the basic evidence that pharmacological inhibition of autophagy resulted in the accumulation of Epstein-Barr Virus nuclear antigen 1, Münz’s group demonstrated that autophagy is indeed implicated in the endogenous processing of viral antigens for MHCII-mediated presentation (Paludan et al., 2005). Blum and collaborators observed that also CMA is involved in the MHC-II restricted presentation of cytosolic glutamic acid decarboxylation antigen (Zhou et al., 2005).

In addition to intracellular antigens, B cells recognize antigens on the surface of neighboring cells through the BCR and have evolved a sophisticated strategy to extract these antigens, internalize and degrade them for presentation to T cells. Surprisingly, internalized BCRs were found in structures resembling autophagosomes that transport them toward Toll-like receptor 9-positive (TLR9^+^) endosomes to optimize BCR signaling in response to nucleic acid antigens (Chaturvedi et al., 2008) or to a compartment enriched in protein arginine deaminase to favor presentation of citrullinated peptides (Ireland and Unanue, 2011). Finally, components of the autophagic machinery have been recently implicated in proper centrosome repositioning during immune synapse formation, which is instrumental for the polarized trafficking of the BCR and lysosomes to the contact area, where immobilized antigens are acquired and processed for presentation (Arbogast et al., 2019).

## p66Shc Is a New Regulator of B Cell Autophagy and Differentiation

### p66SHC Is a Pleiotropic Regulator of B Cell Mitophagy

**FIGURE 3:**
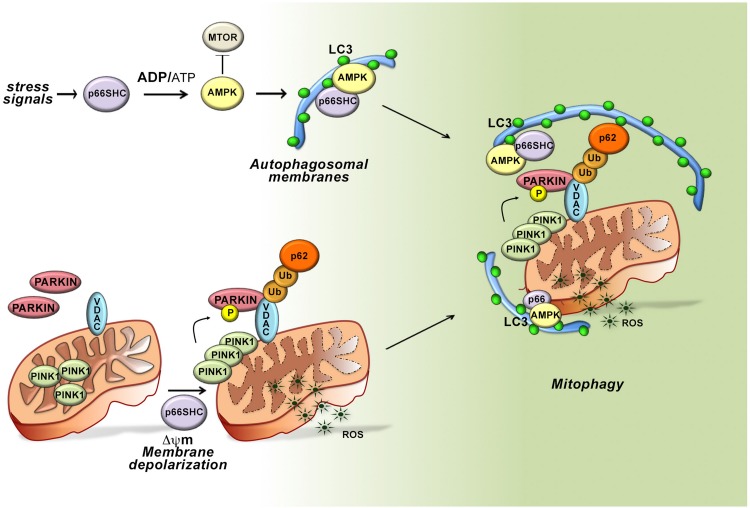
Role of p66SHC-dependent autophagy/mitophagy in B cells. In the presence of p66SHC, B cells undergo oxidative stress with insufficient ATP production and metabolic imbalance. The increased ADP/ATP ratio activates AMPK and enhances the autophagic flux. p66SHC impairs ATP production not only by modulating the AMPK pathway but also by dissipation of the mitochondrial membrane potential and ROS generation. The loss of mitochondrial integrity activates the PINK1-PRKN/PARKIN-mediated mitophagy pathway and increases the ubiquitination of OMM proteins, such as the Voltage-Dependent Anion Channel (VDAC) protein. The ubiquitinated proteins are recognized by the autophagic adaptor SQSTM1/p62 that mediates the recruitment of phagophore membranes through LC3 interaction. p66SHC is also able to interact with membrane-associated LC3 through an LC3-interacting region (LIR) motif and form a complex with active AMPK. Although the mitochondrial pool of p66SHC is localized in the intermembrane space, ubiquitination of OMM proteins causes OMM rupture, making mitochondrial p66SHC accessible for interaction with LC3-II to promote phagophore membrane recruitment to damaged mitochondria. Hence, p66SHC impacts cellular metabolism and mitochondrial function in B cells acting as an LC3 receptor to recruit autophagosomal membranes to dysfunctional mitochondria primed to be removed via mitophagy.

We have recently reported that p66SHC affects B cell survival not only by antagonizing survival signaling by the BCR and promoting apoptosis, but unexpectedly also through selective autophagy/mitophagy (Onnis et al., 2018). Based on the ability of p66SHC to inhibit glycolysis in fibroblasts, which leads to a decrease in ATP production (Soliman et al., 2014), we asked whether p66SHC modulates metabolism in B cells. Using the p66Shc-deficient B cell line MEC1 (Capitani et al., 2012; Cattaneo et al., 2016) transfected with a p66SHC-encoding vector, or B cells isolated from *p66Shc*^–/^*^–^* and wild-type mice (Migliaccio et al., 1999), we found that p66SHC negatively impinges on cellular metabolism. Compared to p66SHC-deficient B cells, p66SHC-expressing B cells showed a limited ATP production due to reduced glycolysis and mitochondrial function. Indeed, in the presence of p66SHC a reduction in the glycolytic intermediates pyruvate, lactate and citrate was observed, concomitant with a lower mitochondrial transmembrane potential. The resulting imbalance in the ATP:AMP ratio led to the activation of the energy sensor AMPK at the expense of the nutrient sensor mTOR (Figure 3). Consistent with the link between this metabolic status and autophagy (He and Klionsky, 2010; Egan et al., 2011; Russell et al., 2014), we found that p66SHC promotes B cell autophagy.

To dissect the molecular circuitry that connects p66Shc to autophagy we searched for the molecular determinants within its sequence that are implicated in this function. Interestingly, we identified three putative LC3 interacting region (LIR) motifs, which bind the membrane-associated form of LC3 (LC3-II). Of these, one is localized in the unique N-terminal CH2 domain that characterizes this SHC1 isoform and was thus potentially relevant to p66SHC-dependent autophagy. The pro-autophagic activity of p66SHC was indeed abrogated when the LIR motif was mutated, highlighting the potential of p66SHC to function as an LC3-II adaptor. Immunoprecipitation experiments on MEC1 transfectants expressing either wild-type p66SHC or its LIR mutated version showed that p66SHC interacts with LC3-II through the putative LIR motif. p66SHC was also found to exploit the LIR motif to interact with the active form of AMPK. In agreement with the fact that LC3-II forms at cell membranes as the result of LC3 cleavage and lipidation (Tanida et al., 2004), this complex was predominantly associated with cell membranes.

The ability of p66SHC to promote the intracellular accumulation of ROS is also expected to contribute to the metabolic imbalance and hence to its pro-autophagic activity. We therefore extended the analysis to p66SHC mutants lacking either S36 in the CH2 domain or E132-133 in the CYCS binding domain. Disrupting CYCS binding selectively abrogated the ability of p66SHC to promote autophagy, indicating that two distinct interactions, mediated by the CYCS binding motif and the LIR motif, respectively, are required for p66SHC-mediated autophagy. Hence p66SHC promotes autophagy through three distinct yet interconnected activities: (i) by impacting cellular metabolism; (ii) by disrupting mitochondrial integrity; and (iii) by functioning as an LC3-II receptor.

The dual ability of p66SHC to induce dissipation of the mitochondrial membrane potential and to interact with LC3-II suggests the possibility that its pro-autophagic activity may be exploited to eliminate damaged mitochondria through their targeting to phagophores and eventual mitophagy. Indeed, we found that p66SHC-expressing cells undergo enhanced mitophagy in the presence of mitochondrial uncouplers, which induce mitophagy. Of note, while both the CYCS and LIR interacting motifs were required for p66SHC-dependent mitophagy, mitochondrial membrane depolarization was only dependent on CYCS binding, suggesting that dissipation of the mitochondrial transmembrane potential and the resulting local ROS production was essential for the pro-mitophagic activity of p66SHC upstream of LC3-II recruitment.

Depolarized mitochondria are targeted for selective autophagy through ubiquitylation of proteins at the outer mitochondrial membrane (OMM) by the ubiquitin ligase PARKIN, which is phosphorylated and stabilized at this location by the kinase PINK1 (Matsuda et al., 2010). Ubiquitylated proteins are recognized either by the autophagy-initiating component ULK1 that is phosphorylated by AMPK, or by autophagy receptors that recruit preformed phagophores through their interaction with LC3-II (Matsuda et al., 2010). Interestingly, we found that mitochondria were hyperubiquitylated in p66SHC-expressing B cells, concomitant with an increase in the mitochondrial association of active AMPK, the autophagosome-nucleating complex components BECLIN-1 and VPS34, and LC3-II. Additionally, p66SHC was able to form a complex with LC3-II and active AMPK at mitochondria. These data highlight p66SHC as a new regulator of autophagy that it promotes by disrupting mitochondrial activity, thereby marking damaged mitochondria through their ubiquitylation, and additionally by acting as a receptor to recruit to mitochondria both preformed phagophores and the autophagy-initiating kinase AMPK. A conundrum in this scenario is that the mitochondrial pool of p66SHC resides in IMS, which would preclude its interaction with LC3-II. Interestingly, PHB2, a protein associated with the inner mitochondrial membrane, has been a recently shown to also act as an LC3-II receptor, similar to p66SHC. This property requires proteasome-mediated degradation of the OMM (Tanaka et al., 2010; Chan et al., 2011; Yoshii et al., 2011). We found that the integrity of the OMM is impaired in the presence of p66SHC. This finding, taken together with the fact that proteasome inhibitors prevent the association of p66SHC with LC3-II and AMPK, suggest that OMM degradation, primed by p66SHC-dependent depolarization, sets the conditions for IMS -associated p66SHC to interact with LC3-II to promote mitophagy (Figure 3).

It is intriguing that p66SHC can promote both apoptosis and mitophagy, with one molecular determinant, the CYCS binding site on which its ROS-elevating activity depends, shared for both functions, and the LIR motif solely required for mitophagy. This highlights p66SHC as a molecular hub that senses intracellular signals, driving the cell toward apoptosis or autophagy depending on the metabolic or stress status. This dual and opposite function is shared by other proteins, such as p53 and PARKIN1 (Chen et al., 2013; Carroll et al., 2014). Interestingly, these sensors of cell stress have cytoprotective (mitophagy) or cytotoxic (apoptosis) modes, depending on the degree of mitochondrial damage. Hence p66SHC may function as molecular switch, determining which pathway to activate to help B-cells prolong survival (autophagy) or alternatively die by apoptosis when the levels of intracellular ROS and mitochondria damage increase beyond a critical threshold.

### p66SHC Controls B Cell Differentiation by Promoting Hypoxia-Induced Mitophagy

Interestingly, we found p66SHC promotes B cell mitophagy also in response to hypoxia. In B cells, hypoxia enables cells to adapt to a range of oxygen tensions as they develop, migrate and differentiate. During their differentiation in GCs, B cells are governed by a hypoxic microenvironment. Additionally, mitochondrial ROS are key determinants in cell fate regulation, with ROS^*high*^ B cells undergoing CSR and ROS^*low*^ cells differentiating to plasma cells (Jang et al., 2015). Hence, the pro-mitophagic and pro-oxidant functions of p66SHC suggest a role for this protein adaptor in B-cell differentiation. Consistent with this notion, we demonstrated that B cell differentiation to plasma cells was enhanced in the absence of p66SHC, with a concomitant reduction in CSR. We previously demonstrated that p66SHC deficiency does not affect B-cell development while enhancing BCR-induced proliferation in mature B cells (Capitani et al., 2010). These new findings highlight a wide-ranging implication of p66SHC in peripheral B cell fate.

## Concluding Remarks

p66SHC emerges as an important regulator of B cell survival and differentiation that functionally connects the pathways responsible for cell fate and fitness *via* autophagy/mitophagy or apoptosis. In physiological conditions ROS act as intracellular signaling molecules, with mitochondria representing the main source of ROS (Karihtala and Soini, 2007; Marchi et al., 2012). Excessive ROS production leads to a state of oxidative stress that has been implicated in the pathogenesis of a number of malignancies, including breast, lung, liver, and prostate cancers (Moller et al., 2008; Khandrika et al., 2009; Nowsheen et al., 2009; Severi et al., 2010; Adcock et al., 2011; Sakaguchi et al., 2011; Vera-Ramirez et al., 2011; Thapa and Ghosh, 2012). On the other hand, abnormally high ROS levels lead to apoptosis, and, thus, inhibit both tumor initiation and progression. As a quality control pathway, autophagy prevents tumor initiation and progression via suppression of oxidative and genotoxic stress through removal of damaged or aggregated proteins and organelles, which are known promoters of ROS production. However, autophagy may also support the survival, metabolism and growth of tumoral cells under nutrient-sufficient conditions (Guo et al., 2011; White, 2012). By functioning as a rheostat in cell commitment to apoptosis or autophagy, p66SHC may play an important role both in maintaining cell homeostasis under physiological growth conditions and in fine-tuning cell viability under stress. It should be underscored that the expression of p66SHC can be modulated in response to both physiological and pharmacological stimuli (Migliaccio et al., 1999; Patrussi et al., 2018). Additionally, the pro-oxidant activity of p66SHC is regulated post-translationally by S36 phosphorylation, a process strongly affected by cellular stress (Migliaccio et al., 1999; Nemoto and Finkel, 2002; Pinton et al., 2007). Since both expression levels and phosphorylation status contribute to the metabolism-related function of p66SHC (Soliman et al., 2014), these factors are likely to have a major impact on its pro-autophagic function. Interestingly, we have previously reported that p66SHC expression is severely impaired in CLL B cells, a defect that contributes *via* multiple pathways to the abnormally extended survival of these cells (Capitani et al., 2010; Patrussi et al., 2019). This pathological setting is ideal to address the molecular mechanisms coordinated by p66SHC that contribute to leukemogenesis through the functional interplay between ROS and autophagy. For example, it could be hypothesized that damaged mitochondria fail to be eliminated in the p66SHC-deficient CLL B cells, thereby contributing to genomic instability and leukemia progression. The metabolism-related function of p66SHC may also provide a valuable cue to understanding the metabolic abnormalities of CLL B cells, which have been largely associated with mitochondrial alterations (see Galicia-Vazquez and Aloyz, 2019). These considerations warrant further studies to elucidate the role of p66SHC in the control of B cell homeostasis and the relevance of its dysregulation to leukemia.

## Author Contributions

AO, CC, FF, and CB wrote the review. AO, FF, and CC prepared the artwork.

## Conflict of Interest

The authors declare that the research was conducted in the absence of any commercial or financial relationships that could be construed as a potential conflict of interest.
